# Metagenomic next-generation sequencing unveils invasive aspergillosis masquerading as miliary tuberculosis in a neutropenic leukemia patient: a case report

**DOI:** 10.3389/ffunb.2026.1751760

**Published:** 2026-03-27

**Authors:** Hong L. Ji, Cong H. Liu, Cheng X. Nie, Jia F. Luo, Xin R. Li, Ai S. Fu, Yan L. Ge

**Affiliations:** 1 North China University of Science and Technology School of Clinical Medicine, Tangshan, Hebei, China; 2 Department of Endocrinology, North China University of Science and Technology Affiliated Hospital, Tangshan, Hebei, China; 3 Department of Respiratory Medicine, North China University of Science and Technology Affiliated Hospital, Tangshan, Hebei, China

**Keywords:** acute monocytic leukemia, case report, invasive pulmonary aspergillosis, metagenomic next-generation sequencing (mNGS), miliary tuberculosis

## Abstract

**Background:**

Empirical anti-tuberculosis therapy is a common strategy when patients with acute leukemia chemotherapy-induced neutropenia develop diffuse pulmonary small nodular and subsolid lesions. However, the absence of pathogenetic verification may lead to catastrophic consequences.

**Methods:**

Following negative conventional microbiological cultures (bronchoalveolar lavage bacterial culture, Gram/Gram-negative test) and ineffective anti-infective therapy, a second bronchoscopy revealed caseous obstructive lesions in the right upper lobe bronchus. Metagenomic Next-Generation Sequencing (mNGS) analysis of lavage fluid ultimately confirmed invasive pulmonary fungal disease.

**Results:**

The mNGS analysis of the bronchoalveolar lavage fluid (BALF) reported 6,750 *Aspergillus fumigatus* sequences, 43 *Aspergillus* complex sequences, and 81 *Candida albicans* sequences (considered airway colonization with no pathogenic significance), confirming probable invasive pulmonary aspergillosis (IPA) in line with the 2023 revised EORTC/MSGERC consensus criteria for invasive fungal diseases. Following discontinuation of anti-tuberculosis therapy, targeted antifungal treatment with amphotericin B (40 mg daily) was initiated. Post-treatment, the patient’s temperature normalized. Follow-up CT demonstrated improved absorption of lesions in the left lung and right lower lobe, with stable cavitary nodules in the right upper lobe.

**Conclusion:**

This case demonstrates that invasive pulmonary fungal infection can perfectly mimic the typical radiographic features of hematogenous disseminated pulmonary tuberculosis, including diffuse small nodular and subsolid lesions with a miliary distribution pattern predominantly in the upper lobes and extrapulmonary manifestations such as erythema nodosum. For unexplained pulmonary infections in immunocompromised hosts where conventional diagnosis and empirical treatment fail, the timely application of bronchoscopy combined with mNGS technology represents a critical breakthrough for achieving precise diagnosis.

## 1 Introduction

The incidence of invasive fungal infections (IFI) in patients with hematological malignancies has increased substantially, with rates varying across countries (Pagano et al., 2006, 2017; Gamaletsou et al., 2018). Opportunistic fungal pathogens are the primary causative agents, chiefly comprising *Candida*, *Aspergillus*, and *Cryptococcus* species (Badiee and Hashemizadeh, 2014). Multiple risk factors contribute to IFI in leukaemia patients, including neutropenia, steroid use, high-dose chemotherapy, foreign medical devices, genetic predisposition, advanced age, and comorbidities (Ali et al., 2006; Chaudhri et al., 2020). The period of granulocytopenia following chemotherapy represents a high-risk phase for IFI in leukaemia patients. Sites of IFI infection may range from superficial to systemic involvement. However, respiratory tract infections are most common, with the lungs being the most frequent target organ, presenting with diverse radiographic manifestations. Miliary nodular shadows are highly suggestive of hematogenous disseminated tuberculosis but may also be caused by other pathogens (e.g., fungi, viruses, other bacteria). Although aspergillosis is common in immunocompromised hosts, its typical presentation involves halo signs and cavities. Diffuse miliary nodular patterns are relatively uncommon and may be easily confused with tuberculosis.

This case report describes a patient with acute myeloid leukaemia who developed pulmonary lesions mimicking miliary tuberculosis following chemotherapy. After a protracted diagnostic journey, the condition was ultimately confirmed as invasive fungal pulmonary disease (IFPD). This experience serves as a critical warning to clinicians in hematology, infectious diseases and related fields: pulmonary infections in immunocompromised patients exhibit high dynamism and superimposed characteristics. Treatment response and radiological changes must be reassessed in conjunction with pathogenetic evaluation. Meanwhile, this case also highlights the critical value of metagenomic next-generation sequencing (mNGS) in the etiological diagnosis of culture-negative, empirically treated ineffective difficult pulmonary infections in immunocompromised hosts, which has been widely used in clinical practice in China and globally in recent years.

## 2 Case report

This section is structured and reported in full compliance with the CARE Case Report Guidelines, covering the complete clinical course, diagnostic reasoning, and treatment process of the case.

### 2.1 Patient information

A de-identified 45-year-old Han Chinese male was admitted to our hospital on November 21, 2023, with a 3-month history of leukopenia, accompanied by intermittent high fever (peak 39.0°C) and tender erythema nodosum on the bilateral lower extremities. Three months before admission, the patient developed unexplained bilateral lower limb erythema nodosum accompanied by fever (peak 39.0 °C). Blood tests at an external hospital revealed pancytopenia (WBC 1.75 × 10^9^/L, HGB 88 g/L, PLT 132 × 10^9^/L) with 56% immature cells in peripheral blood differential. He had no history of chronic infectious diseases, hematological malignancies, hereditary disorders, or drug/food allergies, with no relevant family history of infectious or hematological diseases. The patient had stable social support and maintained full compliance with all medical advice during hospitalization. His primary clinical concern was persistent fever and progressive pulmonary lesions after chemotherapy-induced severe neutropenia.

### 2.2 Clinical findings and diagnostic assessment

On admission, the patient’s vital signs were: body temperature 38.2°C, heart rate 98 beats/min, respiratory rate 22 breaths/min, blood pressure 125/78 mmHg, and room air oxygen saturation 96%. Physical examination only noted 1–3 cm tender erythematous subcutaneous nodules on the extensor surfaces of bilateral lower limbs, with no superficial lymphadenopathy or positive signs in cardiopulmonary, abdominal, and neurological examinations. Post-admission tests confirmed leukopenia with mild anemia, 50% immature cells in peripheral blood, and 69% primitive immature monocytes on bone marrow morphology, establishing a definitive diagnosis of acute monocytic leukemia (AML-M5). Gene testing further identified adverse prognostic FLT3/ITD and IDH1 mutations, confirming high-risk disease.

**Figure 1:**
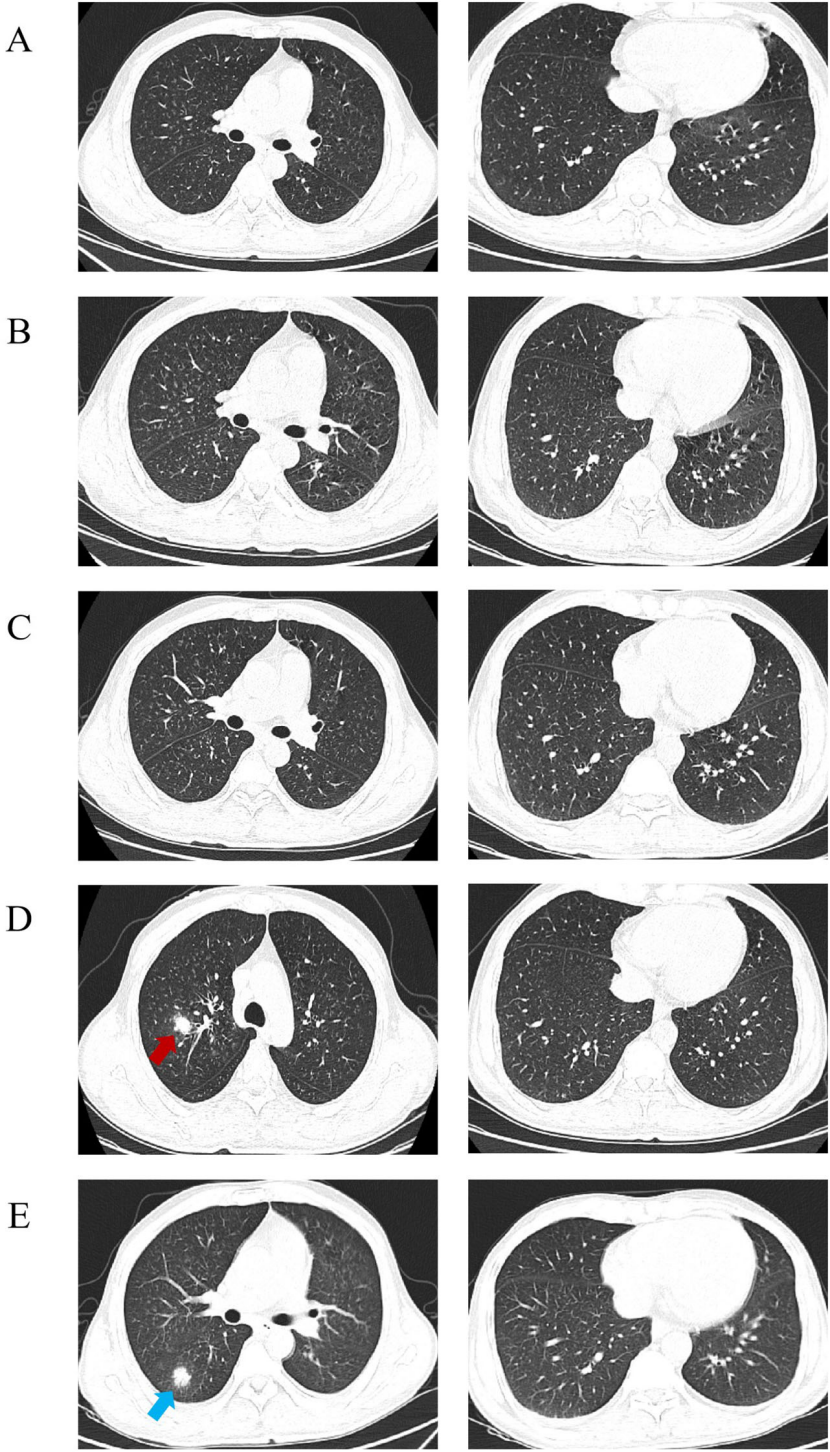
Evolution of serial chest CT images during the patient’s treatment. **(A)** (2023-11-27): Initial chest CT before anti-infective therapy revealed diffuse small nodular and subsolid lesions with a miliary distribution pattern in both lungs, predominantly in the upper lobes, which was the imaging basis for initial differential diagnosis. **(B)** (2023-12-02): Follow-up after doxycycline therapy showed a slight increase in the number of nodular lesions in both lungs, indicating no response to initial anti-infective therapy. **(C)** (2023-12-10): Following treatment with moxifloxacin and the first bronchoscopy, nodular shadows in the left upper lobe showed slight reduction, though other lesions persisted, leading to expert consultation for further diagnosis. **(D)** (2023-12-22): On the 7th day of empirical anti-tuberculosis therapy, follow-up CT showed increased number and size of nodular shadows in the right upper lobe (indicated by arrows), suggesting disease progression, which was the key turning point for the decision to perform the second bronchoscopy + mNGS testing. **(E)** (2024-01-02): On the 8th day of targeted antifungal therapy with amphotericin B, follow-up CT revealed stabilization of the cavitary nodule in the right upper lobe(indicated by arrows), with significant absorption of lesions in the left lung and right lower lobe, confirming the effectiveness of targeted therapy.

Induction chemotherapy with azacitidine plus venetoclax was initiated on November 24, 2023. The patient had intermittent fever (36.9–39.0 °C) after chemotherapy, and developed severe prolonged neutropenia, with a nadir absolute neutrophil count of 0.08×10^9^/L on December 14. Serial chest computed tomography (CT) was performed to evaluate the etiology of fever: chest CT on November 27 (**Figure 1A**) showed multiple miliary nodules in bilateral lungs, predominantly in the upper lobes; repeat chest CT on December 2 (**Figure 1B**) showed progressive increase in miliary nodules; follow-up chest CT on December 10 (**Figure 1C**) showed no significant regression of pulmonary nodules.

Conventional microbiological tests and empirical treatment were performed sequentially for etiological diagnosis: empirical doxycycline was first administered for suspected Mycoplasma pneumoniae infection, with no sustained improvement in fever. Doxycycline was then switched to intravenous moxifloxacin, and the patient’s temperature normalized temporarily. Bronchoalveolar lavage (BAL) was performed on December 4, which showed no bacterial growth, with negative cryptococcal antigen. Blood tests on December 14 showed severe pancytopenia (WBC 0.7 × 10^9^/L, NEU 0.08 × 10^9^/L, HGB 61 g/L, PLT 96 × 10^9^/L), with negative G and GM tests. The etiology of the lung lesions remained unclear; remote infectious disease consultation was performed, and hematogenous disseminated tuberculosis was suspected given the clinical presentation and positive PPD test.

**Figure 2:**
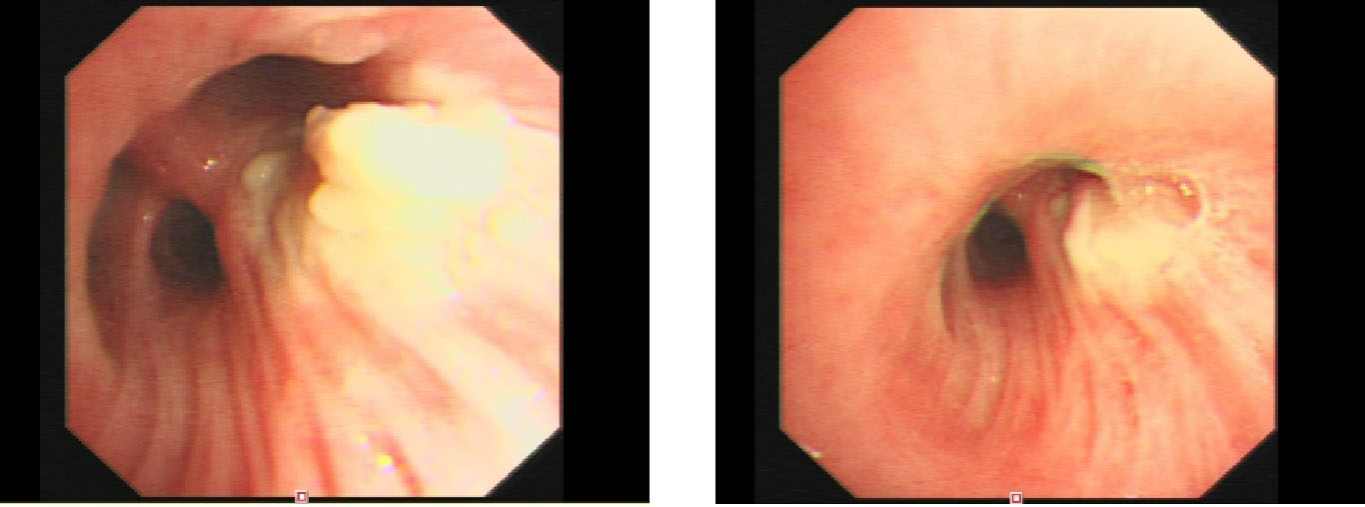
Bronchoscopic findings during treatment. Bronchoscopic findings of airway invasive lesions during the second bronchoscopy (2023-12-25). The image shows the posterior segment of the right upper lobe nearly obstructed by copious caseous necrotic material (indicated by arrow), which is direct endoscopic evidence of *Aspergillus* airway invasion, and the key basis for confirming invasive infection and performing mNGS testing.

Standard anti-tuberculosis therapy plus linezolid and low-dose prednisolone was initiated on December 15. The patient’s fever subsided briefly, but recurrent high fever occurred on December 20. Repeat chest CT on December 22 (**Figure 1D**) showed further progression of pulmonary nodules. Despite combined anti-tuberculosis, broad-spectrum antibacterial (meropenem), and empirical escalated amphotericin B therapy, the patient’s fever persisted. A second bronchoscopy with BAL was performed on December 25 (**Figure 2**), with caseous necrotic tissue nearly obstructing the right upper lobe bronchus on endoscopy, and sampling targeting the left lower lobe posterior basal segment (the most active lesion with an accessible airway for safe sampling). Metagenomic next-generation sequencing (mNGS) of BAL fluid detected 6,750 reads of *Aspergillus fumigatus*, 43 reads of *Aspergillus* complex, and 81 reads of *Candida albicans*.

In accordance with the 2023 revised EORTC/MSGERC consensus criteria, a definitive diagnosis of probable invasive pulmonary aspergillosis (IPA) was established. Core diagnostic evidence included: (1) Host factor: severe prolonged neutropenia post-chemotherapy; (2) Clinical features: progressive pulmonary nodular lesions with bronchial caseous necrosis; (3) Mycological evidence: high-abundance *A. fumigatus* sequences in BALF mNGS. The low-abundance *C. albicans* was judged as airway colonization with no pathogenic significance. Differential diagnoses of disseminated tuberculosis, bacterial pneumonia, and leukemic pulmonary infiltration were ruled out sequentially.

### 2.3 Therapeutic interventions

Standard anti-tuberculosis therapy was discontinued immediately after the definitive IPA diagnosis. Targeted antifungal therapy with intravenous amphotericin B 40 mg daily was continued. This regimen was selected over first-line voriconazole for the following reasons: its broad-spectrum antifungal coverage, better hepatic safety in the setting of chemotherapy-induced liver function impairment, continuity of prior empirical treatment, and local clinical accessibility. Daily monitoring of renal function and electrolytes was performed for treatment safety, and the dosage was adjusted dynamically based on the patient’s renal function and clinical response.

### 2.4 Follow-up and outcomes

After 14 days of targeted amphotericin B therapy, the patient’s body temperature remained continuously normal, and respiratory symptoms were significantly relieved. Follow-up chest CT on January 2, 2024 (**Figure 1E**) showed partial regression of pulmonary lesions, with a small cavity in the right upper lobe nodule. No severe adverse events related to antifungal treatment occurred, and the patient had good tolerance to the regimen.

For the underlying hematologic disease, the patient was diagnosed with high-risk refractory AML-M5. He failed to achieve hematologic remission after multiple lines of induction chemotherapy and developed severe prolonged myelosuppression with transfusion dependence. In April 2024, the patient developed altered mental status and focal neurological deficits, with imaging highly suggestive of central nervous system leukemia. Given the patient’s critical condition, persistent myelosuppression, and uncontrolled systemic disease, the patient’s family opted for palliative care and voluntary discharge.

### 2.5 Case discussion and implications

**Figure 3:**
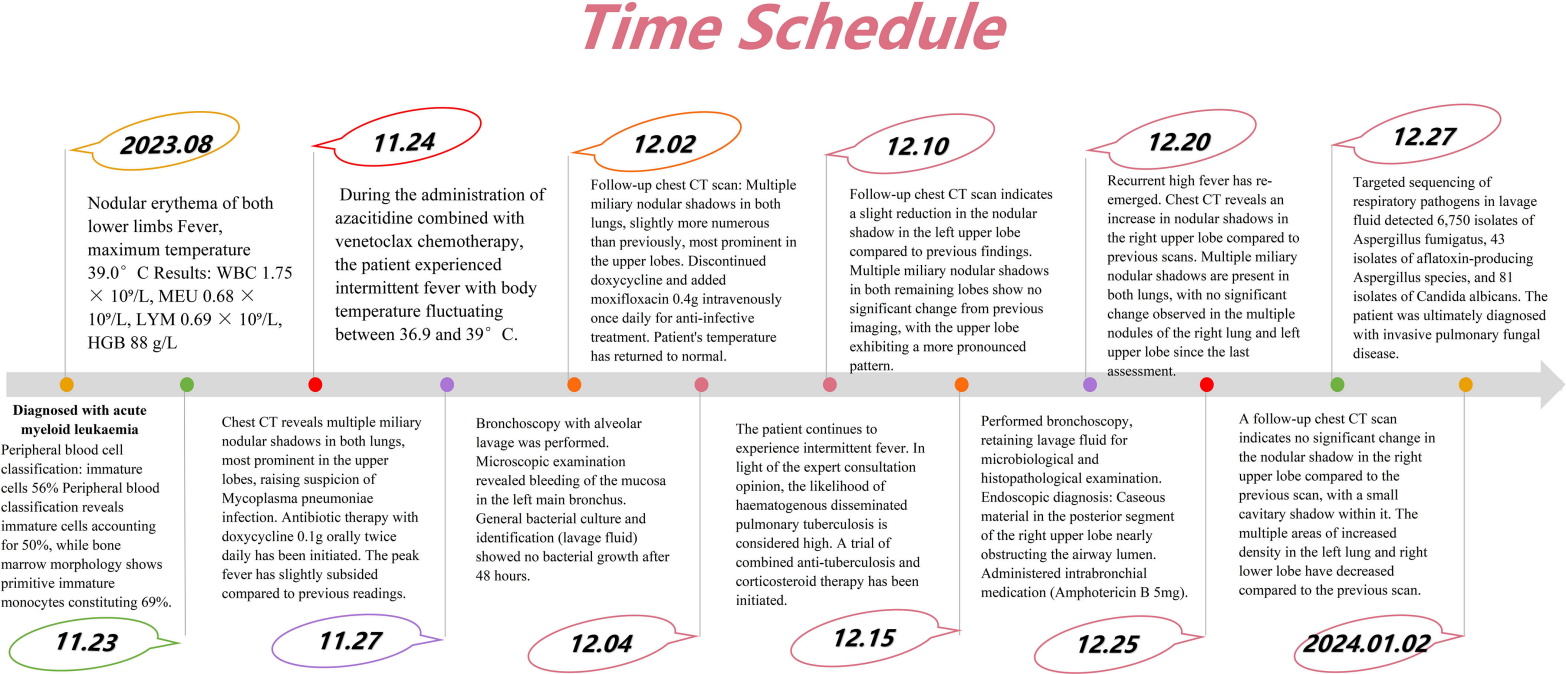
Timeline of patient management and disease progression. This timeline clearly illustrates the entire process from initial admission diagnosis, initiation of chemotherapy, pulmonary imaging changes, sequential anti-infective/anti-tuberculosis therapies, key diagnostic results, to final confirmation and commencement of targeted antifungal treatment. BALF, bronchoalveolar lavage fluid; mNGS, metagenomic next-generation sequencing.

This case highlights the high mortality risk of invasive fungal disease in patients with refractory hematologic malignancies, as well as the diagnostic challenges of IPA in immunocompromised hosts with atypical clinical and radiological presentations. The full timeline of disease progression, diagnostic workup and therapeutic interventions is summarized in **Figure 3**.

## 3 Discussion

Invasive pulmonary aspergillosis (IPA) is a leading cause of infection-related mortality in neutropenic patients with acute leukemia, yet its atypical clinical and radiographic phenotypes frequently lead to diagnostic delay and misdiagnosis (Zhang et al., 2017). The core challenge of this case is the near-perfect mimicry of hematogenous disseminated pulmonary tuberculosis by IPA, which created a critical diagnostic pitfall for the clinical team.

The patient’s initial presentation—persistent fever, upper lobe-predominant diffuse small nodular and subsolid lesions, positive tuberculin skin test (PPD), and bilateral lower limb erythema nodosum—aligned closely with classic disseminated tuberculosis. Erythema nodosum was diagnosed via dermatological consultation based on typical tender, symmetrical lower extremity subcutaneous nodules; biopsy was deferred due to severe thrombocytopenia and neutropenia. This extrapulmonary manifestation was attributed to a delayed hypersensitivity reaction to invasive *Aspergillus* infection, consistent with prior reports of erythema nodosum as an atypical IPA presentation in immunocompromised hosts. Active tuberculosis was definitively ruled out via negative acid-fast bacilli smear and mycobacterial culture of BALF, with the positive PPD test explained by latent tuberculosis infection (LTBI).

Radiological misdiagnosis is a major barrier to timely IPA management, with a reported clinical misdiagnosis rate of 64.29% (Marom and Kontoyiannis, 2011). We identified key radiological distinctions between the patient’s fungal lesions and classic miliary tuberculosis nodules, critical for differential diagnosis in neutropenic hosts. Classic miliary tuberculosis nodules demonstrate the “three uniformities”: uniform 1–3 mm size, uniform density, and symmetric diffuse bilateral distribution, with minimal morphological change over 1–2 weeks (Baratella et al., 2025). In contrast, this patient’s fungal lesions displayed “three non-uniformities”: variable 2–5 mm size, mixed solid/subsolid density with ill-defined margins, asymmetric upper lobe-predominant distribution, and rapid progression to nodule fusion, enlargement, and cavitation within 7 days. These nuanced differences were initially overlooked, compounded by negative serum and BALF (1,3)-β-D-glucan (G) and galactomannan (GM) tests, which reinforced the diagnostic bias toward tuberculosis and prompted empirical anti-tuberculosis therapy with systemic corticosteroids.

The negative G/GM test results, a key driver of diagnostic delay, were explained by three patient-specific factors: severe prolonged neutropenia (absolute neutrophil count <0.1×10^9^/L for >10 days) reduced fungal antigen release below the detection threshold; prior empirical low-dose amphotericin B inhibited fungal growth and antigen shedding; and the airway-invasive, non-angioinvasive IPA phenotype limited systemic antigen entry, a setting where serum G/GM test sensitivity is only 30–50%. This case highlights a critical warning: negative fungal antigen tests cannot rule out IPA in neutropenic patients, and over-reliance on these results may cause fatal diagnostic delay.

The 10-day course of 30 mg daily prednisolone during empirical anti-tuberculosis treatment exacerbated IPA progression. Corticosteroids impair neutrophil and macrophage antifungal activity, inhibit Th1-type immune responses, upregulate *Aspergillus* virulence gene expression, and mask disease progression via transient fever reduction, ultimately leading to pulmonary tissue necrosis and cavitation in this case (Xu et al., 2025).

Despite 10 days of standard anti-tuberculosis therapy, the patient developed recurrent high fever and progressive radiological deterioration, prompting a second bronchoscopy. Endoscopy revealed caseous necrotic material nearly obstructing the right upper lobe bronchus, direct evidence of airway tissue invasion. mNGS of BALF from the active infection site detected 6,750 reads of *Aspergillus fumigatus*, confirming a diagnosis of probable IPA per the 2023 revised EORTC/MSGERC consensus criteria, supported by a neutropenic host factor, progressive cavitary pulmonary lesions, and high-abundance mycological evidence in BALF.

Due to non-specific clinical manifestations and the inherent limitations of conventional testing, IPFD is frequently misdiagnosed in immunocompromised hosts with hematological malignancies (Mudge et al., 2005). Conventional diagnostic methods, including microbial culture, serum G/GM tests, and microscopy, have well-documented shortcomings in this population: blood cultures have a positivity rate of <15% for IPA, while serum fungal antigen tests are prone to false-negative results in neutropenic patients with prior antifungal exposure, as seen in this case (Chen et al., 2020). These limitations highlight the critical need for novel molecular diagnostic tools for IPFD. mNGS, a culture-independent broad-spectrum molecular tool, overcomes these barriers by simultaneous detection of all pathogen classes (Miao et al., 2026). It is now widely integrated into global clinical practice, recommended in international guidelines for culture-negative infections in immunocompromised patients, and is a core diagnostic method for refractory pulmonary infections in Chinese tertiary hospitals. Key pitfalls remain, including false results from prior antimicrobial use or environmental contamination, and an inability to directly distinguish pathogenic infection from colonization.

For mNGS interpretation, we applied an international standard multi-dimensional framework to differentiate infection from colonization (Zhao et al., 2025): (1) microbial load (high fungal reads >1000 support pathogenicity); (2) tissue invasion evidence (endoscopic/imaging necrotic lesions); (3) clinical correlation (symptom progression aligned with detection, and improvement after targeted therapy); (4) specimen type (BALF from the active infection site); (5) host immune status (*Aspergillus* is a classic opportunistic pathogen in neutropenic hosts). Based on this framework, the 6,750 sequence reads of *Aspergillus fumigatus* detected in BALF mNGS, combined with the endoscopic evidence of bronchial caseous necrotic lesions, progressive pulmonary cavitation, and rapid clinical improvement after targeted anti-*Aspergillus* therapy, confirmed its role as the definitive pathogenic agent of invasive infection. In contrast, the 81 reads of *Candida albicans* detected in the same BALF specimen were judged to be benign airway colonization with no pathogenic significance: the patient had no clinical, laboratory, or imaging evidence of invasive candidiasis, no candidemia in multiple blood cultures, and achieved complete symptom resolution with anti-*Aspergillus* monotherapy, with no targeted anti-*Candida* treatment administered.

mNGS was not performed during the first bronchoscopy due to high initial tuberculosis suspicion supported by specialist consultation, transient fever resolution with moxifloxacin, clinical/insurance norms designating mNGS as a second-line tool, and insufficient awareness of atypical miliary IPA. Retrospectively, earlier mNGS would have identified the pathogen 3 weeks earlier, avoiding unnecessary anti-tuberculosis therapy and steroid exposure, and enabling earlier targeted antifungal treatment.

While most current clinical research on mNGS for IFD diagnosis remains in the form of case reports and small-sample observational studies (Chen et al., 2021; Cui et al., 2022), this case provides strong real-world evidence for the clinical value of mNGS in the diagnosis of atypical IPA. In this patient, conventional blood cultures, fungal antigen tests, and microbial cultures were all negative, and mNGS analysis of BALF directly identified a high load of *Aspergillus fumigatus*, providing decisive etiological evidence to confirm the diagnosis. This case clearly demonstrates the significant advantages of mNGS in the rapid, unbiased, and comprehensive detection of pathogens in difficult-to-diagnose infections, particularly for fastidious fungi with low detection rates in conventional tests, in immunocompromised hosts.

This study has the following limitations that need to be acknowledged: (1) Single-case nature: this is a single-center single-case report, and the proposed diagnostic strategy needs to be verified by multi-center large-sample studies to confirm its universality; (2) Lack of histopathological gold standard: percutaneous lung biopsy was not performed due to the patient’s severe thrombocytopenia and neutropenia, and only bronchial mucosal biopsy was performed, without lung parenchymal tissue pathological evidence to confirm parenchymal invasion of *Aspergillus*; (3) Potential overinterpretation of mNGS findings: mNGS can only detect pathogen nucleic acid but cannot distinguish live from dead bacteria or directly confirm pathogenicity, and there is a potential risk of overinterpretation despite comprehensive clinical judgment; (4) Other limitations: differential cell count and cytological examination of BALF were not performed, and *Aspergillus*-specific IgG serological test was not completed; long-term follow-up and intervention for the patient’s latent tuberculosis infection were not performed; the patient’s written perspective was not formally collected, only clinical interview content was recorded.

The diagnostic and therapeutic process of this IPFD case highlights several clinically significant points: (1) For hematological patients with post-chemotherapy neutropenia presenting with diffuse miliary pulmonary nodules, IFI (particularly aspergillosis) must be considered as important differential diagnoses, even when clinical manifestations are highly suggestive of tuberculosis. (2) Response to empirical anti-tuberculosis therapy serves as a crucial diagnostic test. Persistent fever or radiographic deterioration after 1–2 weeks of treatment warrants high suspicion of misdiagnosis, necessitating active investigation of alternative etiologies to avoid diagnostic delay. (3) When conventional microbiological tests yield negative or inconclusive results, molecular diagnostic techniques such as mNGS should be considered early to enhance pathogen detection rates, particularly in complex infections involving immunocompromised hosts. (4) For critically ill patients with neutropenia, fever, and progressive pulmonary lesions, clinical decisions often necessitate broad coverage. However, timely and accurate pathogen identification remains fundamental to achieving targeted therapy and improving prognosis.

In summary, this case describes an atypical IPA presentation mimicking miliary tuberculosis in a neutropenic leukemia patient, with definitive diagnosis confirmed via bronchoscopy and BALF mNGS. It highlights the underrecognized diagnostic pitfalls of atypical IPA in immunocompromised hosts and underscores the critical value of early mNGS application in refractory culture-negative pulmonary infections in this population.

## Data Availability

The original contributions presented in the study are included in the article/supplementary material. Further inquiries can be directed to the corresponding author.

## References

[B1] Ali R. Ozkalemkas F. Ozcelik T. Ozkocaman V. Ozkan A. Bayram S. . (2006). Invasive pulmonary aspergillosis: role of early diagnosis and surgical treatment in patients with acute leukemia. Ann. Clin. Microbiol. Antimicrob. 5, 17. doi: 10.1186/1476-0711-5-17, PMID: 16872530 PMC1550418

[B2] Badiee P. Hashemizadeh Z. (2014). Opportunistic invasive fungal infections: diagnosis & clinical management. Indian J. Med. Res. 139, 195–204. 24718393 PMC4001330

[B3] Baratella E. di Luca V. Oliva A. Fiorese I. Segalotti A. Troian M. . (2025). CT imaging features of pulmonary sarcoidosis: typical and atypical radiological features and their differential diagnosis. Med. (Mex.) 61, 2094. doi: 10.3390/medicina61122094, PMID: 41470097 PMC12734719

[B4] Chaudhri E. Fathi W. Hussain F. Hashmi S. K. (2020). The increasing trends in cases of the most common cancers in Saudi Arabia. J. Epidemiol. Glob. Health 10, 258–262. doi: 10.2991/jegh.k.200515.001, PMID: 32959621 PMC7758845

[B5] Chen L. Su Y. Xiong X.-Z. (2021). Rhizopus microsporus lung infection in an immunocompetent patient successfully treated with amphotericin B: A case report. World J. Clin. cases 9, 11108–11114. doi: 10.12998/wjcc.v9.i35.11108, PMID: 35047625 PMC8678878

[B6] Chen P. Sun W. He Y. (2020). Comparison of the next-generation sequencing (NGS) technology with culture methods in the diagnosis of bacterial and fungal infections. J. Thorac. Dis. 12, 4924–4929. doi: 10.21037/jtd-20-930, PMID: 33145066 PMC7578456

[B7] Cui Q. Dai H. Wu D. He J. Xu Y. Tang X. . (2022). Case report: A case of acute T lymphoblastic leukemia with mixed infection of lethal invasive mucormycosis and multi-drug resistant bacteria. Front. Med. 9. doi: 10.3389/fmed.2022.854338, PMID: 35479945 PMC9037592

[B8] Gamaletsou M. N. Walsh T. J. Sipsas N. V. (2018). Invasive fungal infections in patients with hematological Malignancies: emergence of resistant pathogens and new antifungal therapies. Turk. J. Hematol. 35, 1–11. doi: 10.4274/tjh.2018.0007, PMID: 29391334 PMC5843768

[B9] Marom E. M. Kontoyiannis D. P. (2011). Imaging studies for diagnosing invasive fungal pneumonia in immunocompromised patients. Curr. Opin. Infect. Dis. 24, 309–314. doi: 10.1097/QCO.0b013e328348b2e1, PMID: 21673574

[B10] Miao Y. Bai D. Fan S. Li H. Yao J. Mo J. . (2026). Liquiritigenin alleviates inflammation and mitochondrial dysfunction in acute pancreatitis via ERβ–VDAC1 signaling. J. Ethnopharmacol. 354, 120526. doi: 10.1016/j.jep.2025.120526, PMID: 40912483

[B11] Mudge D. W. Johnson D. W. Isbel N. M. Campbell S. B. Nicol D. L. Hawley C. M. (2005). Obliterative bronchiolitis or opportunistic infection? A diagnostic challenge in a renal transplant patient. Nephrol. Dial. Transplant. Off. Publ. Eur. Dial. Transpl. Assoc. - Eur. Ren. Assoc. 20, 246–247. doi: 10.1093/ndt/gfh580, PMID: 15632365

[B12] Pagano L. Busca A. Candoni A. Cattaneo C. Cesaro S. Fanci R. . (2017). Risk stratification for invasive fungal infections in patients with hematological Malignancies: SEIFEM recommendations. Blood Rev. 31, 17–29. doi: 10.1016/j.blre.2016.09.002, PMID: 27682882

[B13] Pagano L. Caira M. Candoni A. Offidani M. Fianchi L. Martino B. . (2006). The epidemiology of fungal infections in patients with hematologic Malignancies: the SEIFEM-2004 study. Haematologica 91, 1068–1075. 16885047

[B14] Xu J. Wang B. Ao H. (2025). Corticosterone effects induced by stress and immunity and inflammation: mechanisms of communication. Front. Endocrinol. 16. doi: 10.3389/fendo.2025.1448750, PMID: 40182637 PMC11965140

[B15] Zhang R. Chen J. Huang H. Ma J. Meng F. Tang Y. . (2017). Primary fungal prophylaxis in acute leukemia patients with different risk factors: retrospective analysis from the CAESAR study. Int. J. Hematol. 106, 221–228. doi: 10.1007/s12185-017-2224-2, PMID: 28390035

[B16] Zhao J. Zhuge R. Hu B. Wang Y. Wang X. Zhang Y. . (2025). Clinical impact of bronchoalveolar lavage fluid metagenomic next-generation sequencing in immunocompromised patients with severe community-acquired pneumonia in ICU: a multicenter retrospective study. Infection 53, 1911–1927. doi: 10.1007/s15010-025-02520-0, PMID: 40268850 PMC12460554

